# Spatial-Frequency Feature Learning and Classification of Motor Imagery EEG Based on Deep Convolution Neural Network

**DOI:** 10.1155/2020/1981728

**Published:** 2020-07-20

**Authors:** Minmin Miao, Wenjun Hu, Hongwei Yin, Ke Zhang

**Affiliations:** ^1^School of Information Engineering, Huzhou University, Huzhou 313000, China; ^2^Zhejiang Province Key Laboratory of Smart Management & Application of Modern Agricultural Resources, Huzhou University, Huzhou 313000, China

## Abstract

EEG pattern recognition is an important part of motor imagery- (MI-) based brain computer interface (BCI) system. Traditional EEG pattern recognition algorithm usually includes two steps, namely, feature extraction and feature classification. In feature extraction, common spatial pattern (CSP) is one of the most frequently used algorithms. However, in order to extract the optimal CSP features, prior knowledge and complex parameter adjustment are often required. Convolutional neural network (CNN) is one of the most popular deep learning models at present. Within CNN, feature learning and pattern classification are carried out simultaneously during the procedure of iterative updating of network parameters; thus, it can remove the complicated manual feature engineering. In this paper, we propose a novel deep learning methodology which can be used for spatial-frequency feature learning and classification of motor imagery EEG. Specifically, a multilayer CNN model is designed according to the spatial-frequency characteristics of MI EEG signals. An experimental study is carried out on two MI EEG datasets (BCI competition III dataset IVa and a self-collected right index finger MI dataset) to validate the effectiveness of our algorithm in comparison with several closely related competing methods. Superior classification performance indicates that our proposed method is a promising pattern recognition algorithm for MI-based BCI system.

## 1. Introduction

Brain computer interface (BCI) technology [1–3] uses multiple brain function signals, including scalp Electroencephalogram (EEG) [4], Local Field Potentials (LFPs) [5], and Electrocorticography (ECoG) [6], to establish a direct communication channel between human brain and external devices. This characteristic of BCI is extremely important for patients with severe brain nerve damage, since the normal communication channel for such patients has been blocked [7]. Considering the convenience, safety, and cost, scalp EEG is most frequently used in BCI fields. Among various BCI control paradigms, motor imagery- (MI-) based BCI system is a very important branch. Via MI-based BCI system, users can control robots or external machines merely by movement imagination, without the intervention of peripheral nerve. Due to its great potential application value in motor function rehabilitation [8], motor function assistance, and so forth, MI-based BCI system has been widely concerned.

EEG pattern recognition is an important part of MI-based BCI system; traditional EEG pattern recognition algorithm mainly includes two steps, namely, feature extraction and feature classification. In feature extraction stage, common spatial pattern (CSP) algorithm [9–11] is the most commonly used algorithm, but several factors would affect the performance of CSP algorithm, such as the spatial channels, frequency bands of sensorimotor rhythm signal, and time windows. It is worth noticing that most of the research efforts have been dedicated to optimizing the frequency bands for significant CSP features extraction. Filter band common spatial pattern (FBCSP) algorithm [12] is a benchmark for spatial-frequency feature learning and has been widely applied to MI EEG analysis. More recently, a sparse filter band common spatial pattern (SFBCSP) algorithm [13] has been proposed to select most significant CSP features in multiple frequency bands via sparse regression. In the feature classification stage, many machine learning algorithms, such as linear discriminant analysis (LDA) [14], support vector machine (SVM) [13], and logistic regression (LR) [15], are used to classify different EEG patterns of motor imageries. We notice that some more sophisticated algorithms have been also proposed for MI EEG classification in recent years. Jiao et al. [16] developed a sparse group representation model (SGRM) in which a test sample can be estimated as a linear combination of samples in a composite dictionary matrix composed by CSP features from multiple subjects. Jin et al. [17] introduced a sparse Bayesian extreme learning machine (SBELM) method for MI-related EEG classification by combining the advantages of both extreme learning machine (ELM) and sparse Bayesian learning. As we can see that, for most traditional MI EEG pattern recognition algorithms, feature extraction and feature classification are separated; however, these two stages usually have different objective functions; hence it is easy to cause information loss [18].

The convolutional neural network (CNN) is based on deep learning theory and has been widely used in image recognition [19], speech recognition [20], and other fields. Its main characteristics are weight sharing and local perception so that the number of weight parameters is greatly reduced compared with the ordinary deep neural network. In addition, CNN implements feature learning and classification in the network simultaneously, which is simpler and clearer than the traditional pattern recognition method. Furthermore, less information is lost in this procedure.

In the past few years, deep learning techniques, i.e., deep neural networks, have been investigated to deal with complex brain function signals [21]. In terms of the research on MI EEG, the independent CNN and CNN-based hybrid models are widely used. Lee and Choi [22] proposed obtaining time-frequency representations of EEGs using continuous wavelet transform (CWT) as the input of CNN model. Tabar and Halici [23] applied short time Fourier transform (STFT) method to convert EEG temporal series into 2D images and used CNN and stacked autoencoders (SAE) for MI EEG classification. Uktveris and Jusas [24] used Fast Fourier Transform (FFT) energy map method for CNN and achieved satisfactory results for four-class MI EEG classification. Liu et al. [25] introduced a multiscale deep CNN method to deal with the representation for MI EEG signals. Hartmann et al. [26] investigated how CNN represented spectral features through the sequence of intermediate stages of the network. Wang et al. [27] devised a CNN-based method for MI EEG feature extraction and adopted weak classifier for feature selection. Tan et al. [28] trained a deep neural network with CNN and recurrent neural network (RNN) for the EEG classification task. Yang et al. [29] investigated the classification of multiclass MI EEG signals by augmented CSP (ACSP) features and CNN model. Tang et al. [30] constructed a 5-layer CNN model based on the spatiotemporal characteristics of EEG for MI tasks classification. In this paper, a novel deep learning approach is proposed for classification of MI EEG signal. Unlike all these above works, we do not use any complex algorithm, such as CWT, STFT, FFT, and ACSP, for two-dimensional feature map generation. Besides, different from the work in [30] which studied the spatiotemporal characteristics of MI EEG, this work offers an insight into the spatial-frequency features of MI EEG. Furthermore, rather than exploiting the spatial-frequency characteristics of EEG by FBCSP or SFBCSP, we herein propose learning and classifying the spatial-frequency features of MI EEG simultaneously in a unified CNN framework. Specifically, we convert raw EEG data to image representation by computing the energies of multichannel EEG signals in multiple frequency bands at first. Afterward, a novel multilayer CNN model is designed, and the spatial characteristics of MI EEG are analyzed according to the obtained weight parameters of convolution layers. Finally, with a public dataset and a self-collected right index finger motion imagination dataset, extensive experimental comparisons are carried out between our method and several closely related machine learning algorithms.

## 2. Materials and Methods

### 2.1. Dataset Description

In this study, in order to better evaluate the effectiveness of the proposed algorithm, we used two different datasets for analysis. The first dataset is public BCI competition III dataset IVa, in which EEG signals are collected from five subjects (denoted by aa, al, av, aw, and ay) using 118 electrode amplifier. There are two types of MI tasks in the dataset, namely, right hand MI and right foot MI. The time duration of each MI trial is 3.5 seconds, and the sampling rate is set to 1000 Hz. For each subject, there are 280 MI trials (140 trials for hand MI and 140 trials for foot MI) in total. More details about this dataset can be found at http://www.bbci.de/competition/iii/. The second dataset is right index finger motion imagination dataset (denoted by Finger Dataset) which was collected by us. In this dataset, EEG signals are collected from five subjects (denoted by S1, S2, S3, S4, and S5) using 21 electrode amplifier. There are two types of MI tasks in the dataset, namely, finger movement imagination and rest state. The time duration of each MI trial is 4 seconds, and the sampling rate is also set to 1000 Hz. It should be noted that different subjects in this dataset have different numbers of MI trials. For each subject, the exact numbers of finger movement imagination and rest state trials are denoted by the following: subject (finger movement imagination, rest state) S1 (58, 58), S2 (59, 48), S3 (52, 63), S4 (56, 57), and S5 (62, 39). For the BCI competition III dataset IVa, we selected the first 260 trials as the training set, the following 10 trials as the testing set, and the last 10 trials as the validation set. For this public dataset, to more reliably evaluate the classification performance, we also implemented 10-fold cross-validation to compute the average classification accuracy. In each fold of 10-fold cross-validation, each part was for testing and the remaining nine parts were for training (90%) and validation (10%). For Finger Dataset with small sample size, we randomly selected about 80% of the samples as the training set and the remaining samples for testing and validation. The numbers of training samples, validation samples, and testing samples are denoted by S1 (92, 12, 12), S2 (85, 11, 11), S3 (93, 11, 11), S4 (89, 12, 12), and S5 (81, 10, 10).

### 2.2. Methods

#### 2.2.1. EEG Data Representation Transform

Before applying CNN, we firstly convert raw EEG data to image representation. For Finger Dataset, since the sensorimotor rhythm usually appears in the frequency bands of 8–14 Hz (*μ* rhythm) and 18–26 Hz (*β* rhythm), we use bandpass filtering to obtain 8–30 Hz signal component from the original EEG signal. In addition, EEG signals from 0.5 seconds to 2.5 seconds after the appearance of visual cue are usually used for pattern analysis. In this study, the signals in this period are also extracted for subsequent processing. After the above processing, each sample can be represented as a matrix of size 21 × 2000, in which 2000 is the number of sampling points and 21 is the number of electrodes. It should be noted that although the sensorimotor rhythm usually appears in the frequency range of 8–30 Hz, the frequency bands related to MI tasks vary among different subjects, and the optimal frequency band is mostly a local narrow band. In order to learn more precise frequency information, we decompose the EEG signal in the range of 8–30 Hz into 10 subbands, the width of each subband is 4 Hz, and the overlap between adjacent subbands is 2 Hz. After that, each sample can be represented as a matrix of size 21 × 10 × 2000, where 10 is the number of frequency subbands. Then, for the EEG signal in each frequency subband of each spatial electrode, we calculate its signal energy as follows:

(1) $$\begin{array}{c} p=\log\left(\mathrm{var}\left(x\right)\right), \end{array}$$

where var(**x**) is the variance of EEG signal sequence **x**. Thus, each sample can be represented as a matrix of size 21 × 10, and each element of the matrix represents the energy of EEG signal in a certain subband of a certain EEG electrode. For each subject in the dataset, we then normalize the EEG energy as follows:

(2) $$\begin{array}{c} P_{i,j}^{r}=\frac{P_{i,j}^{r}-m_{i,j}}{\delta_{i,j}}, \end{array}$$

where **P** is the energy matrix of each sample, *r* denotes the index of each sample, *m*_*i*,*j*_ denotes the average energy of all samples at this location, and *δ*_*i*,*j*_ is the corresponding standard deviation. After the above steps of signal processing, the original EEG signal is transformed into image representation, in which the EEG electrodes are distributed along the vertical axis and the frequency subbands are distributed along the horizontal axis.

For BCI competition III dataset IVa, the raw EEG data is also bandpass filtered within the range of 8–30 Hz. It should be noted that there are 118 electrodes in this dataset. In order to reduce the burden of subsequent calculation and remove the influence of redundant channels, according to the recommendation of literature [18], we extract the EEG signals from 49 channels for subsequent analysis. After the above processing, each sample can be represented as a matrix of size 49 × 3500, in which 3500 is the number of sampling points and 49 is the number of electrodes. Afterward, frequency domain decomposition, 0.5 to 2.5 seconds' time period segmentation, energy extraction, and normalization processing are also carried out. Then, each sample can be represented as a matrix of size 49 × 10.

**Figure 1:**
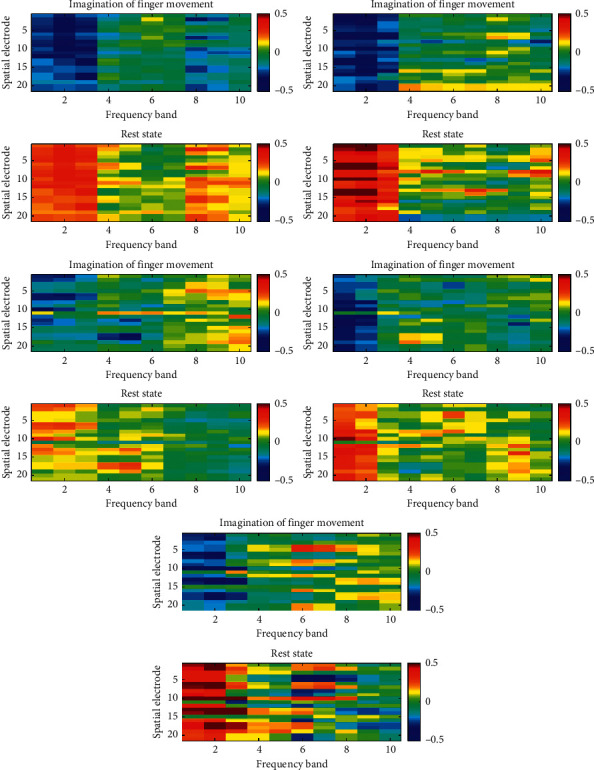
Spatial-frequency energy distributions of EEG in different motor imagery states (Finger Dataset).

**Figure 2:**
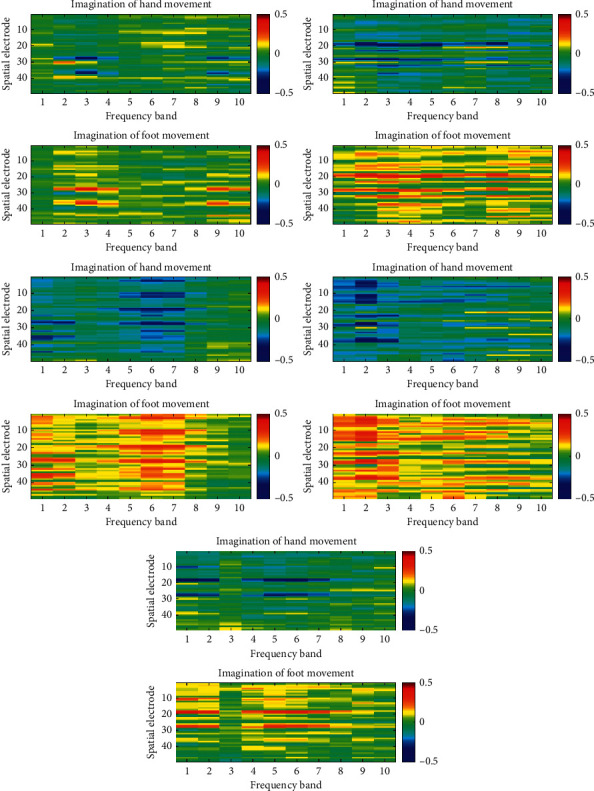
Spatial-frequency energy distributions of EEG in different motor imagery states (BCI competition III dataset IVa).

In order to observe the spatial-frequency characteristics of EEG after preprocessing, for each subject in the two datasets, we divided all samples into two groups according to the categories of MI and calculated the mean value of each group for comparison.  Figure 1 and Figure 2 show the distributions of EEG spatial-frequency energy characteristics of subjects in Finger Dataset and the BCI competition III dataset IVa under different MI states, respectively.

From Figure 1, it can be seen that, compared with the rest state, the energy at the specific electrode decays when finger MI is conducted, so there is a pattern difference in this area, and this phenomenon mainly occurs on the opposite side of the motion imagining limb, such as electrode C3 (index 10); all of the above phenomena conform to the ERD theory [31]. Further observation shows that this pattern difference has obvious frequency domain characteristics; for example, it is most obvious in the 8–14 Hz frequency band. From Figure 2, it can be observed that the energy attenuation of hand motion imagination is more intense than foot motion imagination, so this relatively stable pattern difference appears in several local spatial-frequency blocks. It should be noted that, from Figure 1 and Figure 2, there are different forms of spatial-frequency pattern differences among different subjects. Specifically, the locations of significant spatial-frequency blocks and degrees of differences are different. All these factors will affect the final pattern recognition results. At the same time, we need to focus on whether CNN model can learn spatial-frequency features adaptively.

#### 2.2.2. CNN Structure Design

**Figure 3:**
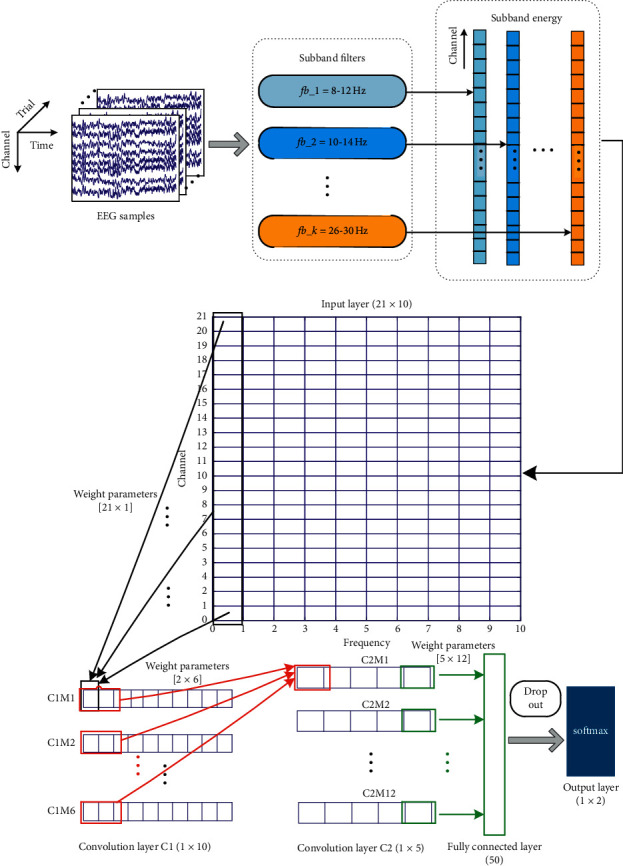
The structure diagram of convolutional neural network for motor imagery EEG pattern recognition (Finger Dataset).

Through the descriptions in the previous section, we can see that the MI EEG signal has a very obvious spatial-frequency characteristic, which is also consistent with the basic ERD theory, and has a relatively stable pattern difference after being converted into image form. In order to learn the MI EEG characteristics adaptively and carry out pattern recognition, this section designs a novel multilayer CNN structure, as shown in Figure 3. The proposed CNN model consists of five layers; the first layer is the input layer which is specially designed to capture the spatial-frequency characteristics of MI EEG signals. Note that we do not use any complex algorithm and retain the energy change information in spatial-frequency domain more completely. The next two layers are the convolution layers, mainly for spatial-frequency feature extraction. It should be noted that we use one-dimensional convolutional filters in the first convolution layer for better analysis of spatial features. The last two layers are the fully connected layer and the softmax layer; these two layers mainly complete the classification task. To prevent overfitting, we implemented dropout regularization before the output layer. The specific description of each layer is as follows.

1. Input layer: the input is the image form of the preprocessed motor imagery EEG sample. For the Finger Dataset, the input is the matrix of size 21 × 10, where 21 is the number of electrodes and 10 is the number of frequency subbands. For BCI competition III dataset IVa, the input is the matrix of size 49 × 10, where 49 is the number of electrodes and 10 is the number of frequency subbands.
2. Convolution layer C1: the main function of this layer is to filter the input signal in the spatial domain (i.e., different electrodes are assigned with different weight values). Therefore, one-dimensional convolution operation (convolution of spatial electrodes on the vertical axis) is carried out in this layer, and the convolution kernel slides along the horizontal axis. This shows the characteristics of CNN, namely, weight sharing and local perception. It should be noted that, since there is no hybrid of any time or frequency domain information, by observing the spatial filter obtained from this layer, we can understand part of the spatial characteristics of motor imagery EEG. In this study, we use 6 spatial filters; thus we can get 6 feature maps after spatial convolution. For the Finger Dataset, the convolution kernel size is set to [21 × 1]. For the BCI competition III dataset IVa, the convolution kernel size is set to[49 × 1]. Since there are 10 frequency subbands, the size of the feature map is [1 × 10].
3. Convolution layer C2: this layer mainly combines the features of the previous layer and learns more complex and abstract spatial-frequency features. The convolution operation of this layer still adopts the concept of local connection and weight sharing. Each convolution operation uses two feature values from all the feature maps of the previous layer and slides along the horizontal axis. The sliding step is also set to two to reduce the number of parameters. In this layer, we set up 12 filters in total, the size of the convolution kernel is [2 × 6], and finally we obtain 12 feature maps of size [1 × 5].
4. Full connection layer: this layer mainly connects the convolution layer C2 and the final output layer. We have set up 50 neurons in this layer. Because of the full connection, each neuron is connected with all the feature values of the previous layer.
5. Output layer: the function of this layer is to output the predicted MI category. Since this study only involves two types of motor imagination, it only contains two neurons, and each neuron is connected with all neurons in the previous layer.

The training of CNN model is realized by back propagation algorithm. Given the input data, all neurons in the network structure produce the activation values according to the initial weight values, bias values, and activation function. According to the output of the softmax layer, the loss function value of the model is calculated. Then, the gradients of the weight and bias terms are computed based on the value of loss function. Afterward, the parameters of the network are updated according to the gradient values. In this study, we initialize the weight value of the network to a random value in the normal distribution with the mean value of 0 and the standard deviation of 0.1 and uniformly initialize the bias value to 0.1. All neurons in front of the output layer use the Rectified Linear Unit (RelU) as the activation function, and its operation is as follows:

(3) y=ReLUwΤx+b=max0,wΤx+b,

where **x** is the feature vector, **w** is the weight value vector, *b* is the bias value, and *y* is the output activation value. Many studies have shown that the traditional sigmoid function has the problem of gradient vanishing. However, using the RelU activation function, which is similar to the neuron response mechanism in the biological neural system, we can usually achieve satisfactory training effect [32]. The output layer adopts the softmax model, and the specific operation of the model is as follows:

(4) $$\begin{array}{c} z_{i}=w^{i}x+b^{i},\left(i=1,2\right),\\ y_{i}=\frac{e^{z_{i}}}{\sum_{j=1}^{2}e^{z_{j}}}. \end{array}$$

Each output value of softmax model represents the probability that the sample belongs to a certain category, and the category with the maximum value is the category of the final output. The loss function uses cross entropy (CE):

(5) $$\begin{array}{c} H\left(p,q\right)=-\sum_{x}p\left(x\right)\ln\left(q\left(x\right)\right), \end{array}$$

where *p* and *q* are the probability distributions of the predicted and original categories, respectively.

Gradient descent method is used to update weight and bias terms, and the optimizer adopts adaptive Adam algorithm. Note that, in order to prevent overfitting and achieve better results in the test set, the dropout [33] operation is performed before the output layer in this study. When training the neural network, some neurons are discarded according to a certain probability *P*(*d*), and the model is trained according to the sparse network structure. In the testing stage, all the neurons are used, but all the weights are corrected to 1-*P*(*d*) times of the original weights. In this study, we set *P*(*d*) to 0.5.  Table 1 provides an overview of the CNN model hyperparameters.

## 3. Experiments

**Figure 4:**
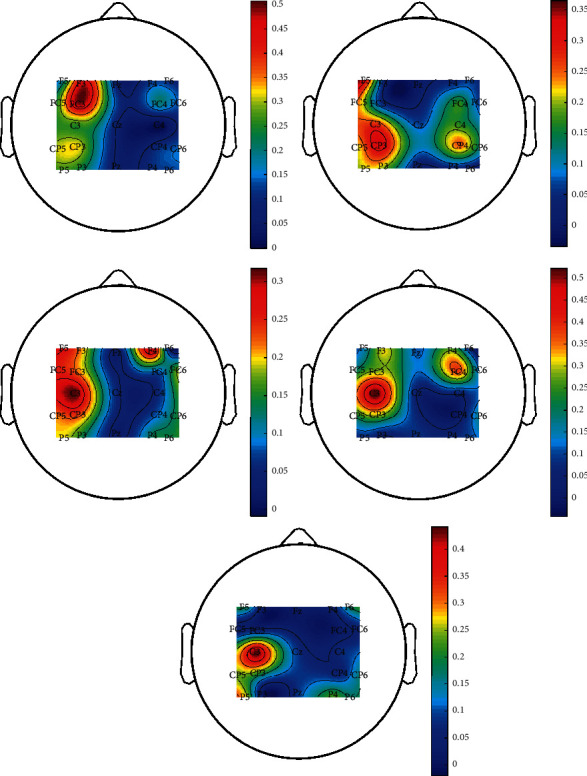
Spatial filter brain pattern distribution (Finger Dataset).

As mentioned in Section 2, the function of the first convolution layer is mainly to filter the original input signal in the spatial domain. According to the theory of ERD, the brain area most related to motor imagery is usually located on the opposite side of the motor imagery limb. In order to intuitively understand the characteristics of the spatial filter learned by CNN, for the Finger Dataset, we extract a filter from each subject's six filters for brain topographic map illustration.  Figure 4 shows the brain pattern distribution of each spatial filter for five subjects in this dataset. It can be seen from the figure that the electrode with the largest weight is usually distributed near the “C3” channel; in another word, the EEG signal in this area has the greatest impact on pattern recognition. This area is just on the opposite side of the right index finger and is in the primary motor cortex. Therefore, the first convolution layer of CNN can learn the spatial characteristics of motor imagination EEG well. It should be noted that the learning of the above parameters is completely without manual operation, which is the result of network parameter updating and iteration, so CNN shows strong adaptive learning ability.

**Figure 5:**
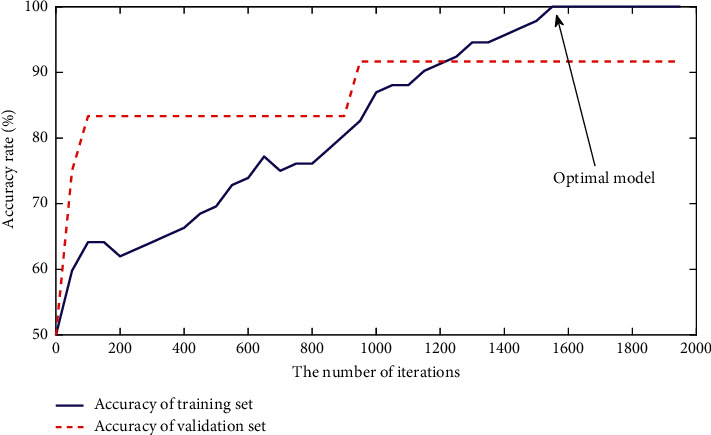
Classification accuracy of validation set and training set during CNN training (subject S1 of Finger Dataset).

In this study, we train CNN model according to training set and validation set. After training, the models of all the subjects can converge effectively. Take subject S1 in the Finger Dataset as an example, the change curve of classification accuracy is shown in Figure 5, the blue solid line shows the classification accuracy of the training set, the red dotted line shows the classification accuracy of the validation set, the abscissa shows the number of iterations, and the ordinate shows the classification accuracy. It can be seen from Figure 5 that, after 1600 iterations, the classification accuracy of training set reaches the highest value and then remains stable, while the classification accuracy of validation set has been kept at the highest value, so it can be considered that the model achieves the best training effect after 1600 iterations, and the trained model is considered as the optimal classification model of subject S1.

In this study, we evaluate the classification performance of CNN model according to the classification accuracy of test set. The software and hardware platforms of the proposed CNN model are Intel (R) Core (TM) i5-8500 3.00 GHz CPU, 8.0 GB RAM, Spyder, Python 3.6, and TensorFlow 2.0 (CPU). With two EEG datasets, extensive experimental comparisons are carried out between our method and other closely related approaches.

For BCI competition III dataset IVa, we firstly used the fixed sample set segmentation for classification performance evaluation, namely, 260 samples for training, 10 samples for testing, and another 10 samples for validation. To evaluate the classification performance of our method objectively, three other algorithms including CSP [9], FBCSP [12], and SFBCSP [13] were adopted for performance comparison. Note that CSP is the baseline method for MI EEG pattern recognition; FBCSP and SFBCSP are state-of-the-art methods for MI EEG spatial-frequency feature learning, which are closely related to our method. The experimental settings for these competing methods are listed as follows.

- CSP : EEG signals on 49 channels were extracted as suggested in [18]. EEG data between 0.5 and 2.5 s after the visual cue were used for feature extraction. A bandpass filter with passband of 8 to 16 Hz has been applied to capture the *μ* rhythm [17]. The number of CSP filters was set to 2 [13]. Three widely used machine learning algorithms, including LDA, SVM, and LR, were used for classification.
- FBCSP : EEG signals on the same 49 channels as in CSP were extracted. EEG data between 0.5 and 2.5 s after the visual cue were used. 6 bandpass filters having bandwidth of 6 Hz in the range of 4 to 40 Hz with no overlap have been used, as described in [34]; these settings gave optimal results. Mutual information-based feature selection has been performed as it gave the best results in [35]; SVM was used for classification.
- SFBCSP : EEG signals on the same 49 channels as in CSP were extracted. EEG data between 0.5 and 2.5 s after the visual cue were used. 17 bandpass filters having bandwidth of 4 Hz with an overlap of 2 Hz in the range of 4 to 40 Hz have been used, as in [13]. The regularization parameter *λ* was determined by 10-fold cross-validation, and linear kernel SVM was adopted for classification as in [13].

Table 2 lists the classification accuracies of CSP + LDA, CSP + LDA, CSP + LR, FBCSP, SFBCSP, and our method on BCI competition III dataset IVa. From this table, we can observe that our method achieves the same results or performs better than all competitors for subjects aa, al, and av and gives the highest average accuracy of 90%.

**Figure 6:**
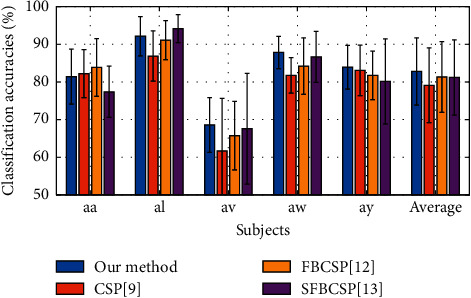
Classification accuracies of 10-fold cross-validations performed by our method and three other competing methods (BCI competition III dataset IVa).

For the public BCI competition III dataset IVa, to more reliably evaluate the classification performance of our method, we further implemented 10-fold cross-validation to compute the average classification accuracy and also compared it with CSP, FBCSP, and SFBCSP. The settings of these three competing methods remained unchanged; however for CSP only SVM was adopted since it gave the best results in Table 2. We also adjusted our CNN model; the changes are mainly in three aspects: (1) 6 bandpass filters having bandwidth of 6 Hz in the range of 4 to 40 Hz with no overlap were used; thus the size of the input matrix was 49 × 6, where 49 was the number of electrodes and 6 was the number of frequency subbands. (2) Two more fully connected layers were added, and the activation functions were changed. (3) The optimizer was changed to Adadelta.  Table 3 summaries the CNN model architecture for 10-fold cross-validation; the adjusted CNN model contains 2 convolution layers, a flatten layer, 3 fully connected layers, and an output layer. Elu and Relu are chosen as activation function for convolution layers and fully connected layers, respectively.  Figure 6 presents classification accuracies derived by CSP, FBCSP, SFBCSP, and our method. The two modified CSP algorithms and our method outperformed the baseline CSP algorithm. Our proposed CNN model further yielded higher average classification accuracy than those of the FBCSP and SFBCSP methods. The average classification accuracy improvements achieved by our method were 3.66%, 1.44%, and 1.59% in comparison with CSP, FBCSP, and SFBCSP methods, respectively.

**Figure 7:**
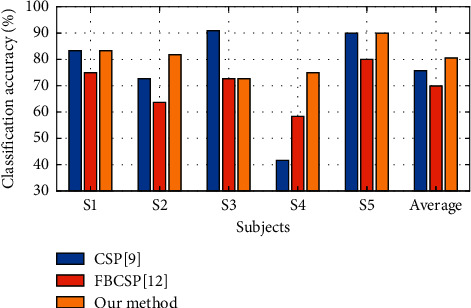
Classification accuracies derived by CSP, FBCSP, and our method (Finger Dataset).

For the Finger Dataset, CSP and FBCSP were adopted for performance comparison. Sparse learning methods usually need more training samples to ensure the performance; thus, we did not use SFBCSP method for this small dataset. Note that, for this dataset, LDA classification algorithm was adopted since it gave superior performance compared to LR and SVM.  Figure 7 presents classification accuracies derived by CSP, FBCSP, and our method for this dataset. Our method yielded higher classification accuracies for most subjects than those of the other two competing methods. The average classification accuracy improvements achieved by our method were 4.84% and 10.63% in comparison with CSP and FBCSP methods, respectively.

**Figure 8:**
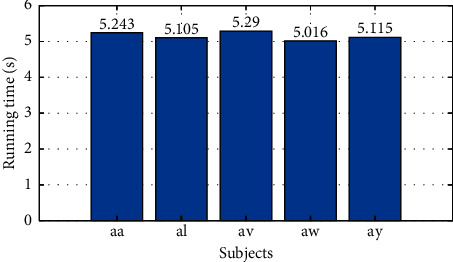
Running times of CNN training for all subjects in BCI competition III dataset IVa.

To further understand the computational cost of the proposed CNN model, for BCI competition III dataset IVa with fixed sample set segmentation, we recorded the training time of each subject (1600 iterations), and Figure 8 summaries the time durations of CNN training for all subjects in this dataset. From this figure we observe that all CNN models can be trained within about 5 seconds, which is an acceptable time cost for real application.

## 4. Discussion

As we can see from Table 2, Figure 6, and Figure 7, overall our algorithm yielded superior classification performance than other competing algorithms. However, we notice that for some subjects, especially the subjects aw and ay in Table 2, the performance of our proposed CNN model is even worse than that of the baseline CSP method. The reasons can be mainly concluded as follows. (1) For all the subjects in a certain dataset, the structures and hyperparameters of the CNN models are the same. However, different users have different characteristics. Therefore, for some subjects the uniform parameter setting may lead to suboptimal solution. (2) The performance of deep learning models highly depends on the size of training data [21]. However, in BCI competition III dataset IVa with fixed sample set segmentation, 260 samples were used for CNN model training; the size of the training set was relatively small, which may affect the stability of the model to some extent. This can be further verified by the results in Figure 6; the classification performances of our method steadily surpass CSP algorithm for subjects aw and ay when 10-fold cross-validations were adopted.

To address the aforementioned limitations and further improve the classification performance of our proposed CNN method, the following three aspects are worthy of our future investigations. (1) Instead of using uniform CNN model parameter setting, we plan to study the characteristics of different subjects and apply user specific CNN structure and parameters for MI EEG pattern recognition. (2) To cope with the problem of small training set and improve the stability of deep CNN model, the combination of deep learning and subject to subject transfer learning is an important research direction in our plan. (3) In this study, the spatial-frequency features of MI EEG were automatically learned by the proposed deep CNN model; however 0.5 seconds to 2.5 seconds after the appearance of visual cue were manually selected. In another word, the features on temporal domain have not been fully studied. As pointed out in [36], the use of a fixed time window could hardly capture discriminative features for all subjects. Based on this, we consider further extending the proposed deep CNN model for MI EEG feature learning on the entire spatial-temporal-frequency domains.

## 5. Conclusion

In this paper, a deep learning algorithm for limb motor imagery EEG pattern classification is proposed. A multilayer CNN model is designed for motor imagery EEG classification, and the spatial-frequency characteristics of motor imagery EEG signals are analyzed according to the obtained parameters of convolution layers in the neural network. Finally, the proposed CNN model is compared with several state-of-the-art machine learning algorithms. In the experiment, the public BCI competition III dataset IVa and a self-collected right index finger movement imagination EEG dataset are used to verify the proposed algorithm. The experimental results demonstrate that the proposed CNN method outperforms all competitors in terms of the mean classification precision for both datasets. In addition, the training time of the proposed model is relatively short. Therefore, we would like to note that the proposed classification method is of great interest for real-life BCI systems.

## Figures and Tables

**Table 1:** Model hyperparameter.

Parameter	Value
Padding	Valid
Optimizer	Adam
Activation function	Relu
Regularization	Dropout
Cost function	Cross_entropy
Batch size	Size of training set

**Table 2:** Comparison of classification accuracy for our method and 5 other competing methods; the highest accuracy is marked in boldface (BCI competition III dataset IVa).

Subject	aa (%)	al (%)	av (%)	aw (%)	ay (%)	Mean (%)
CSP [9] + LR	60	**90**	50	**100**	**100**	80
CSP [9] + SVM	60	**90**	60	**100**	**100**	82
CSP [9] + LDA	60	**90**	50	**100**	**100**	80
FBCSP [12]	60	**90**	60	90	**100**	80
SFBCSP [13]	60	**90**	70	90	**100**	82
Our method	**100**	**90**	**90**	90	80	**90**

**Table 3:** CNN model architecture for 10-fold cross-validation (BCI competition III dataset IVa). Conv refers to convolution layer, Flatten refers to flatten layer, and FC refers to fully connected layer.

	Kernel size	Kernel number	Padding	Activation	Output shape
Conv_1	49 × 1	6	Valid	Elu	1 × 6 × 6
Conv_2	1 × 3	12	Valid	Elu	1 × 2 × 12
Flatten	—	—	—	—	24
FC_1	—	—	—	Relu	50
FC_2	—	—	—	Relu	100
FC_3	—	—	—	Relu	200
Softmax	—	—	—	—	2

## Data Availability

In this study, we used two different datasets for analysis. The first dataset is public BCI competition III dataset IVa and the second dataset is right index finger motion imagination dataset (denoted by Finger Dataset) which was collected by us. For BCI competition III dataset IVa: the BCI competition III dataset IVa used to support the findings of this study has been deposited in the website http://www.bbci.de/competition/iii/. For Finger Dataset: the Finger Dataset used to support the findings of this study is available from the corresponding author upon request.

## Supplementary Materials

Supplementary Materials The supplementary files S1, S2, S3, S4 and S5 corresponds to the five subjects of Finger Dataset, here DynamicSelect corresponds to finger movement imagination, and StaticSelect corresponds to the rest state and all these mat files are the collected MI EEG datasets.
